# Determination of optimal vibration dose to treat Parkinson's disease gait symptoms: A clinical trial

**DOI:** 10.1016/j.prdoa.2024.100248

**Published:** 2024-03-13

**Authors:** Ingrid Pretzer-Aboff, R.K. Elswick, Arnaud Gouelle, Noah Helm, GinaMari Blackwell, Leslie Cloud

**Affiliations:** a Virginia Commonwealth University, School of Nursing, 1100 East Leigh St., Office 3063, Richmond, VA 23298, United States; b Université Reims Champagne Ardenne, UFR STAPS, Laboratory “Performance Santé Métrologie Société”, Reims, France; c Virginia Commonwealth University Health System, Comprehensive Medicine Unit, Richmond, VA 23298, United States; d Virginia Commonwealth University School of Medicine, Department of Neurology, Parkinson’s & Movement Disorders Center, Richmond, VA 23298, United States

**Keywords:** Parkinson’s disease, Vibration, Gait, Freezing of gait

## Abstract

**Introduction:**

Most people with Parkinson's disease (PD) will experience gait problems. Previous studies demonstrated improved gait and balance after vibration stimulation was applied to the feet of PD patients. However, not all study participants showed improvement, perhaps due to sub-optimal vibration stimulus. Thus far, the optimal frequency and amplitude of vibration for mitigating gait dysfunction in PD have yet to be systematically explored. This study aimed to deliver vibration to the feet of 26 people with PD gait disturbances. We hypothesized that a global frequency, amplitude, and minimum duration of vibration therapy are required to improve PD gait issues.

**Methods:**

This was a phase Ib trial to identify optimal vibration parameters. Thirteen participants were recruited at Hoehn & Yahr (H&Y) stage II and 13 participants at stage III. Each group was randomly assigned to different frequency and amplitude settings prescribed by the central composite design methodology. Each participant received vibration for 18 min per walking session, for eight sessions spread over one week.

**Results:**

Results showed an optimal response to treatment for frequency (Hz) and amplitude (mm) of vibration based on the Functional Ambulation Performance score for stages II and III. In the H&Y stage II group, stabilization of outcomes occurred after the 4th treatment. This stabilization was not seen in stage III participants.

**Conclusions:**

A global frequency and vibration amplitude have been identified for treating PD gait disorders. Patients with more advanced disease may require a longer duration of therapy.

## Highlights

• An optimal vibration frequency and amplitude dose was identified.
• Gait metrics improved after four vibration treatments in stage II participants.
• Fatigue likely caused slower gains in gait metrics for stage III participants.

## 1 Introduction

Individuals with Parkinson's disease (PD) will inevitably experience mobility issues, including slowed and shuffling gait. About 60 % will also experience freezing of gait (FoG), arguably the most disabling walking disturbance [1]. A wearable vibration device designed to improve gait, including FoG, may facilitate improved mobility and enhance the quality of life for people with PD. For this study, we used the PDVibe2™ (Resonate Forward, LLC, DE) to deliver continuous vibration to the feet of people with PD-related gait disturbance. Preliminary, uncontrolled studies of a single fixed vibration dose (frequency and amplitude) demonstrated that vibration provided by the PDVibe2^TM^ improved PD gait and balance, resulting in continued motor benefit up to two weeks after the stimulus was turned off [2]. However, positive effects were not seen in all individuals. In this study, we took advantage of the PDVibe2™ feature that allows the vibration and amplitude settings to be independently set to test vibration across a wide range of doses. The selected range of doses was examined in participants classified as Hoehn and Yahr (H&Y) disease stage II and stage III.

This study's primary aim was to identify the optimum vibration parameters (frequency and amplitude) and minimum number of treatment sessions required to improve gait metrics in people with mild to moderate PD. The secondary aim was to assess the safety and tolerability of the device and protocol across a full range of vibration parameters.

## 2 Methods

This study was a single-site, phase Ib response trial conducted with the approval of the Virginia Commonwealth University (VCU) Institutional Review Board. Recruitment occurred at the VCU Parkinson's and Movement Disorder Center and local support groups. Study recruitment started on 4/20/2019 and ended on 10/22/2020.

### 2.1 Vibration device

The PDVibe2™ wearable device is remotely activated, noninvasive, lightweight, and worn on both feet (Fig. 1, Supplement). Two linear actuators were placed on the dorsum of the foot and medial aspect of the malleolus over a thin sock. Previous open-label, uncontrolled studies support the safety and tolerability of the same device used in this study [2], [3]. Demonstrated continued motor benefit effect after turning off the device [2], concerns about habituation with constant sensory stimulation [5], and the knowledge that unpredictable stimulation is associated with increased sensitization [6] informed our decision to provide intermittent “treatments” rather than a continuous vibration.

### 2.2 Participants

Individuals were eligible if they were diagnosed with PD by a neurologist using the UK Brain Bank criteria [7], were on a stable PD medication regimen for three months, were at Hoehn and Yahr (H&Y) stages II or III (while “ON”), could walk independently or with a simple assistive device (e.g., cane, walker), and were observed to have PD-related gait disturbances while they were on PD meds.

Individuals were excluded from the study if they had known Parkinson’s Plus syndrome or if there was evidence of dementia determined by a score of <21 using the Montreal Cognitive Assessment [8]. They were also excluded if they had other disorders impairing gait, stance, balance, or coordination (e.g., stroke, peripheral neuropathy) or used braces or orthotics to ambulate. It is unknown how the vibration device might affect implanted electronics; therefore, individuals with these devices were excluded. Individuals with Deep Brain Stimulation (DBS) devices were included, as previous research demonstrated safety with concurrent use of the PDVibe2™ [3].

### 2.3 Optimal vibration dose and sample size

We used a response surface analysis, an efficient experimental design, to estimate an optimal response to treatment. Details of the method and vibration frequency and amplitudes tested are described in the Supplemental Material.

### 2.4 Randomization

Participants in each H&Y group were randomly assigned to one of nine vibration frequency and amplitude settings as prescribed by the response surface analysis. Our biostatistician (RKE) generated a simple random allocation sequence in numbered sealed envelopes for each H&Y group. After informed consent was obtained, the envelope was opened by the study coordinator (GB), who set the vibration frequency and amplitude accordingly. All other research team members and participants were blinded to the treatment assignment.

### 2.5 Intervention protocol

After the screening/baseline visit, participants received vibration while walking a prescribed path once or twice daily (at least three hours apart) for eight treatment sessions over one week. Participants were on PD medicines for all treatment and evaluation sessions. DBS, if present, remained turned on. Participants were asked to refrain from starting new rigorous exercise routines. DBS settings and PD medications remained the same for the duration of the study, confirmed by self-report.

During each 22-minute vibration treatment session, participants walked back and forth along a 24-foot path, with one direction going over a 20-foot Zeno™ instrumented walkway system (ProtoKinetics, PA). Pre and post-gait measurements were gathered in the initial and final two minutes without vibration. The middle 18 min, with vibration turned on, were divided into three 6-minute walking periods (Fig. 3, Supplement). Two-minute breaks followed each 6-minute walking period to mitigate fatigue, although participants could take additional breaks if necessary. The vibration treatment was turned off during breaks. Participants were monitored for tolerance of the protocol using the Borg Perceived Exertion scale, if they reported “somewhat hard or greater”, they were encouraged to stop and rest. Participants were considered compliant if they participated in at least 80 % of the treatment sessions.

### 2.6 Measures

Baseline data was collected during visit one. The eight vibration treatments started on visit two and ended on visit nine. Questionnaires and performance measures were repeated on visit nine.

We estimated the frequency and amplitude vibration settings that would maximize the Functional Ambulation Performance (FAP) score, our primary outcome. The FAP is used for spatiotemporal gait parameter analysis and is calculated using data collected by the Zeno™ instrumented walkway [4], [9], [10].

Gait velocity and the enhanced Gait Variability Index (eGVI) were also calculated. The gait speed of people with PD has been reported to be between 0.18 and 1.21 m/s. For reference, healthy people in their 60 s have a gait speed between 1.3 and 1.36 m/s [11]. The eGVI differentiates low and high gait variability. For the eGVI, a score closest to 100 is better [12]. The FAP is reflective of the organization of the gait pattern and the eGVI of gait variability [12].

We estimated the frequency and amplitude vibration settings to maximize three balance measures (described in the Supplemental Material).

We also assessed the optimal duration of treatment. For this, we looked at FAP scores after each walking session in both groups for a steady period of stability in scores. For instance, if the FAP score initially improved and stabilized after three treatments, future studies could be shorter than eight sessions. If walking continued to improve by the 8th session, this would similarly inform future studies.

Finally, the safety and tolerability of the device and protocol were verified using qualitative interviews. Adverse events inquiry and inspection of a subject fall diary occurred at each participant visit.

### 2.7 Statistics

A response surface analysis (see Supplemental Material) of optimal vibration frequency and amplitude setting was conducted for all participants and was completed on H&Y stage II and III separately to discern possible differences in dose per the severity of the disease. The analysis was repeated for both groups combined. To assess the optimal number of treatment sessions, we looked at FAP scores after each walking session for both groups for a steady period of stability in scores.

## 3 Results

### 3.1 Participant characteristics

Twenty-six participants completed the study. Participant demographics are found in Table 1. Forty-two percent (11 patients) had deep brain stimulation surgery for their PD. Eighty-eight percent (23 patients) experienced at least one fall in the last six months. Ninety-two percent took levodopa, with 30 % being on levodopa monotherapy. Other PD medication usage among participants was as follows: 38 % amantadine, 54 % dopamine agonists, 12 % MAOB inhibitors, and 12 % COMT inhibitors.

**Table 1:** Participant demographics.

	H&Y Stage II		H&Y Stage III		Combined	
	N = 13	%	N = 13	%	N = 26	%
Sex						
Female	0	0	3	23	3	12
Male	13	100	10	77	23	88
Race						
Asian	1	8	0	0	1	4
Black	1	8	0	0	1	4
Caucasian	10	77	13	100	23	88
Hispanic	1	8	0	0	1	4

	Mean	Std Dev	Mean	Std Dev	Mean	Std Dev

Age	69.6	8.5	69.2	8.2	69.4	8.2

### 3.2 Optimum vibration frequency (Hz) and amplitude (mm)

Table 2 represents the optimal vibration frequency and amplitude that maximized the outcomes tested. First, the optimal frequency and amplitude were determined for each disease stage separately to investigate differences; then, both groups were combined.

**Table 2:** Optimum Frequency/Amplitude Comparison Table: H&Y Stage II, III & Combined at Visit 9.

		H&Y Stage II		H&Y Stage III		Combined	
	Optimization	Frequency/Amplitude	Predicted Optimum	Frequency/Amplitude	Predicted Optimum	Frequency/Amplitude	Predicted Optimum
FAP	Maximize	275/0.55	99.2 (68.6, 100*)	275/0.67	86.8 (71.7, 100*)	275/0.55	90.4 (71.7, 100*)
Gait Velocity cm/s	Maximize	275/0.75	111.4 (44.7, 178.1)	262/0.68	95.5 (68.7, 122.4)	275/0.75	101.7 (67.8, 135.7)
eGVI	Minimize	275/0.75	108.1 (73, 152.1)	275/0.75	106.4 (74.2, 138.6)	275/0.75	107.2 (86.1, 128.4)
BBS	Maximize	275/0.75	53.0 (43.4,62.6)	275/0.64	51.7 (46.3, 57.0)	275/0.75	51.8 (45.9, 57.6)
FES-I	Minimize	275/0.55	16† (16†, 36.3)	275/0.75	23.4 (16†, 40.4)	275/0.55	24.4 (16†, 35.9)
TUG seconds	Minimize	275/0.55	8.1 (0‡, 55.8)	275/0.70	9.1 (0‡, 18.5)	275/0.75	10.9 (0‡, 30.9)

FAP, Functional Ambulation Performance score; eGVI, enhanced Gait Variability Index; BBS, Berg Balance Scale; FES-I, Fall Efficacy Scale – International; TUG, Timed Up & Go.

* FAP maximum score is 100; thus upper 95% confidence limit is restricted to 100. [4,9]

† FES minimum score 16; thus, the predicted minimum and lower 95% confidence limit is restricted to 16. [new reference: N. Dewan, J.C. MacDermid, Fall Efficacy Scale-International (FES-I)] J Physiother. 60 (2014) 60. https://doi.org/10.1016/j.jphys.2013.12.014.

‡ TUG minimum score is 0; thus, the lower 95% confidence limit is restricted to 0. [new reference: D. Podsiadlo, S. Richardson, The "Timed Up & Go”: a test of basic functional mobility for frail elderly persons, J Am Geriatr Soc. 39 (1991) 142–148. https://doi.org/10.1111/j.1532-5415.1991.tb01616.x. ]

The H&Y stage II participants model for FAP yielded a maximum response at the frequency of 275 Hz and amplitude of 0.55 mm. The predicted FAP maximum was 99.2 (95 % CI: 68.5, 100). In the H&Y stage III group, the model for FAP maximum response was 275 Hz and 0.67 mm. The H&Y stage III FAP predicted maximum response was 86.8 (95 % CI: 71.7, 100).

All H&Y stage II participants yielded maximum responses in velocity, enhanced gait variability (eGVI), and balance measures with a vibration frequency of 275 Hz. An amplitude of 0.55 mm optimized the FAP score. An amplitude of 0.75 mm maximized the velocity and eGVI. Balance measures were optimized at either an amplitude of 0.55 mm or 0.75 mm, depending on the measure.

Like H&Y stage II participants, nearly all of the H&Y III group demonstrated optimal scores at a frequency of 275 Hz, except for velocity (262 Hz). Amplitudes among H&Y stage III variables varied between 0.64 mm and 0.75 mm.

The combined model suggests that the FAP, our primary outcome, yielded a maximum response at 275 Hz with an amplitude of 0.55 mm. The velocity, eGVI, and BBS also yielded a maximum response at 275 Hz but with an amplitude of 0.75 mm. Balance measures were optimized at 275 Hz and either 0.55 mm or 0.75 mm, depending on the measure.

### 3.3 Optimum number of treatment sessions

In H&Y stage II, a stabilization of FAP scores was observed after the 4th treatment, indicating that four walking sessions were adequate. In stage III participants, we saw a steady improvement in the FAP score, which may suggest that more than eight vibration treatment sessions are necessary for later-stage Parkinson’s patients.

### 3.4 Adverse events

There were eleven treatment-emergent adverse events (TEAEs) (occurring after the start of the treatment protocol). No serious adverse events occurred. Of the eleven TEAEs, none were thought to be due to the vibration device. Five were thought to be related to the walking protocol. These included three patients who experienced leg pain/cramps, one who experienced lightheadedness, and one who had a fall with related skin abrasion while walking. The remaining were thought to be unrelated to the research protocol.

## 4 Discussion

Vibration has become an increasingly popular therapeutic approach for various symptoms of PD. Despite a lack of randomized clinical trials establishing its efficacy, vibrating devices are being marketed worldwide for this purpose. Scientifically rigorous studies have yet to explore the optimal dosage, body site(s) for application, or duration of therapy. This study is the first that we are aware of that begins to fill these evidence gaps systematically.

We identified a global vibration frequency (275 Hz) within the range (155–296 Hz) we tested in our study. Since many models (Table 2) predicted optimal responses at 275 Hz, the actual global optimal frequency may be higher. To determine the frequency to achieve a global optimal response, future studies should increase the top of the frequency range.

Additionally, the generalizability of the results is limited by the overrepresentation of women and DBS participants compared to the general PD population.

One important factor that we suspect influenced the required number of treatment sessions to stabilize gait metrics is the fatigue effect. This was a rigorous walking protocol, which many patients found draining. The H&Y stage II patients stabilized after just four sessions, but the H&Y stage III patients never did. This is either because they required a longer duration of treatment or because the fatigue effect confounded our outcome assessments. Future studies may require modified walking protocols for more advanced-stage patients.

## 5 Conclusions

Vibration across a wide range of frequencies and amplitudes can be applied to the distal lower extremities of ambulating PD patients by the PDVibe2™ safely and with excellent tolerability. Armed with the safety data from this study of the PDVibe2™, treatment protocol, and identified optimal dosages of 275 Hz and 0.55 mm, we are now poised to perform a randomized controlled trial of vibration therapy. Knowledge gained from this study regarding the potential interference of the fatigue effect in more advanced-stage patients will inform the protocol design.

## 6 Author declaration

All authors have approved of the final article.

All authors have made substantial contributions to all of the following: (1) the conception and design of the study, or acquisition of data, or analysis and interpretation of data, (2) drafting the article or revising it critically for important intellectual content, (3) final approval of the version to be submitted.

I.P-A: Research project conception, organization, design, manuscript writing, review, critique, and final approval of version to be submitted.

R.K.E: Research project conception, organization, design, statistical analysis design and execution, manuscript writing, review, critique, and final approval of version to be submitted.

A.G.: Statistical analysis, manuscript writing, review, critique, and final approval of version to be submitted.

N.H.: Research project organization and execution, manuscript writing, review, critique, and final approval of version to be submitted.

G.B.: Research project organization and execution; manuscript review, critique, and final approval of version to be submitted.

L.C.: Research project conception, design, execution, statistical analysis, manuscript writing, review, critique, and final approval of version to be submitted.

## Funding source

This study was funded by 10.13039/100000864The Michael J. Fox Foundation for Parkinson’s Research, MJFF-008440. This sponsor had no involvement in the preparation of the article, study design, collection, analysis, and interpretation of the data, in the writing of this manuscript, or in the decision to submit the article for publication.

## CRediT authorship contribution statement

**Ingrid Pretzer-Aboff:** Writing – review & editing, Writing – original draft, Validation, Supervision, Resources, Project administration, Methodology, Investigation, Funding acquisition, Formal analysis, Conceptualization. **R.K. Elswick:** Writing – review & editing, Writing – original draft, Visualization, Validation, Supervision, Methodology, Funding acquisition, Formal analysis, Data curation, Conceptualization. **Arnaud Gouelle:** Writing – review & editing, Writing – original draft, Validation, Methodology, Formal analysis, Data curation. **Noah Helm:** Writing – review & editing, Project administration, Methodology, Investigation, Data curation. **GinaMari Blackwell:** Writing – review & editing, Supervision, Project administration, Methodology, Investigation, Data curation, Conceptualization. **Leslie Cloud:** Writing – review & editing, Writing – original draft, Validation, Supervision, Software, Resources, Project administration, Methodology, Investigation, Funding acquisition, Formal analysis, Data curation, Conceptualization.

## Declaration of competing interest

The authors declare the following financial interests/personal relationships which may be considered as potential competing interests: Ingrid Pretzer-Aboff is employed by Virginia Commonwealth University (VCU). She has grants (paid to her institution) from NIH NINDS 1R01NS120560-01, The Michael J. Fox Foundation for Parkinson’s Research, U of Penn NIH/NIA P30 Center, William & Mary College subcontract, and intramural grants from the VCU PMDC and the School of Nursing. She has two patents in process: 1) VCU PRE-20-020 “PDSock” provisional patent application, submitted March 2022, and 2) VCU PRE-21-137F “Recognizing Freezing of Gait and Deploying Vibration to Mitigate Symptoms. U.S., patent App No. 63/391,106, July 21, 2022. She is a founder and has an ownership interest in Resonate Forward, LLC, which provided the device for this study; she follows a VCU conflict of interest management plan.

R.K. Elswick, Jr. is employed by VCU SON. He has grants (paid to his institution) from NIH NIDA R21DA058407-01, NIH NINDS R01NS120560-01, VCU Presidential Research Quest Fund, and NIH NCI R01 NR020633. He has no other disclosures.

Arnaud Gouelle is employed by Université Reims Champagne Ardenne, UFR STAPS, Laboratory “Performance Santé Métrologie Société,” Reims, France, and has no other disclosures.

Noah Helm is employed by VCUHS and has one patent in process; VCU PRE-20-020 “PDSock,” provisional patent was submitted in March 2022. He has no other disclosures.

GinaMari Blackwell is employed by VCU. She has no other disclosures.

Leslie Cloud has been a consultant for Abbvie. She has served on an advisory board and speakers bureau for Kyowa Kirin. She is employed by VCU/VCUHS. She has clinical trial contracts (paid to her institution) with Bukwang, Cerevel, and Intra-Cellular Technologies. She has received honoraria from Medlink Neurology, HMP Global, M3 global research team, and Qessential research. She has grants (paid to her institution) from NIH NINDS 1R01NS120560-01, The Michael J. Fox Foundation for Parkinson’s Research, the Virginia Catalyst Fund, and the Parkinson’s Foundation. She has two inventions (Gastrointestinal Symptoms in Neurodegenerative Disease (GIND) scale, VCU Office of Technology Transfer # CLO-11-0R67 and Recognize and Deploy Vibration for Mitigation of Freezing of Gait, VCU Office of Technology Transfer provisional patent #PRE-21-137F (322203-8130), application filed July 2022.
