# Supplementary material for: Determination of optimal vibration dose to treat Parkinson's disease gait symptoms: A clinical trial
Source: Clin Park Relat Disord. 2024 Mar 13;10:100248. doi: 10.1016/j.prdoa.2024.100248 (PMC10973134; doi:10.1016/j.prdoa.2024.100248)
Supplement: Supplementary data 1 [file mmc1.docx]

**Supplemental Material**

**Figure 1. PDVibe2TM - Vibration Device**

**
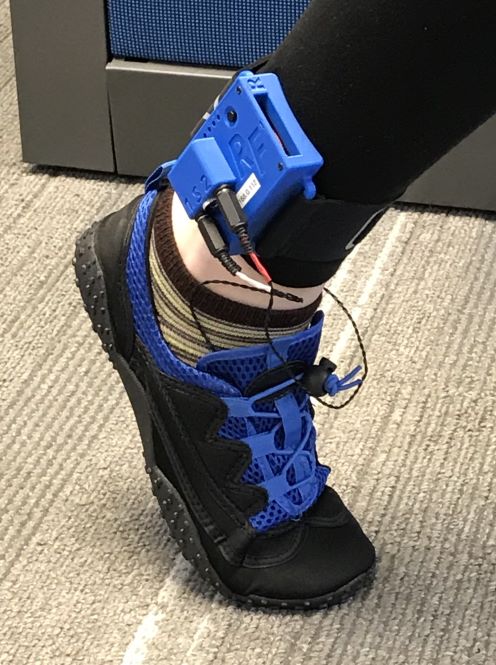
**

**Figure 2. Specification of optimal Amplitude and Frequency using the CCD.**


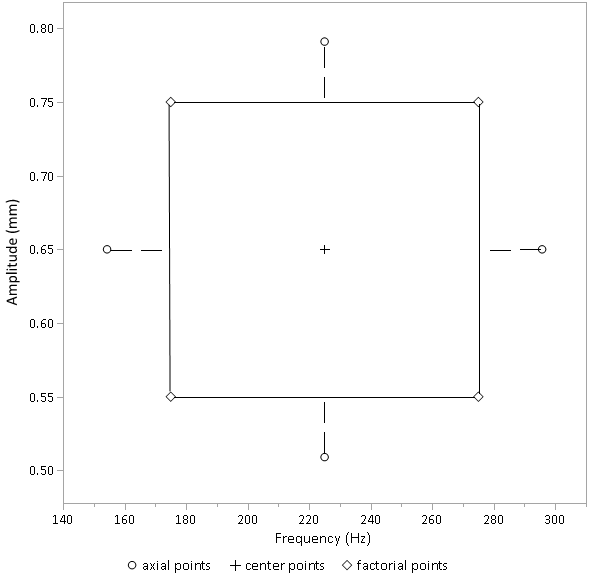


**Response Surface Analysis**

The response surface methodology we utilized was the Central Composite Design (CCD) [1]. The CCD is specified by first defining a 2 x 2 factorial with four (frequency in Hz, amplitude in mm) design points: (175, 0.55), (175, 0.75), (275, 0.55), and (275, 0.75) denoted by the open diamond shape in Figure 2. The design points were based on frequency and amplitude settings used in previous vibration research using this technology [2,3]. The four factorial design points are sufficient to estimate a flat first-order response surface (no curvature). To estimate a second-order response surface (or curvature in the response surface), the 2 x 2 factorial is augmented with axial points and multiple center points. Assuming a frequency and amplitude range of 175 to 275 and 0.55 to 0.75, respectively, from the factorial portion, the axial points are defined as (154.29, 0.65), (295.71, 0.65), (225, 0.509), and (225, 0.791) denoted by the open dots in Figure S1. These axial points are chosen such that the design is rotatable, i.e., the prediction variance is constant on spheres around the center of the design. The number of center points (225, 0.65) (denoted by "X") is chosen to be five to optimize the estimate of the prediction variance and lack of fit. Thus, the full CCD with five center points will require 13 PD participants for each H&Y Stage.

**Figure 3.** The walking protocol for each vibration treatment session was identical, except the vibration settings varied according to the randomization. Everyone received vibration. Rest periods, baseline, and post-treatment two-minute walks were without vibration.


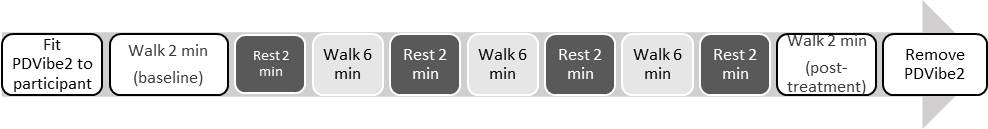


**Balance Measures**

To assess participants balance, we utilized the Fall Efficacy Scale – International (FES-I) [4], the Berg Balance Scale (BBS) [5], and the Timed Up & Go (TUG) test [6].

References

[1] R.H. Myers, D.C. Montgomery, C.M. Anderson-Cook, Response surface methodology: process and product optimization using designed experiments, Fourth edition, Wiley, Hoboken, New Jersey, 2016.

[2] R. Aggarwal, I. Pretzer-Aboff, K. Winfree, S. Agrawal, M. Behari, Clinical outcomes of step-synchronized vibration training in patients of Parkinson’s disease with freezing of gait, Ann Mov Disord. 2 (2019) 15. https://doi.org/10.4103/AOMD.AOMD_18_18.

[3] K.N. Winfree, I. Pretzer-Aboff, D. Hilgart, R. Aggarwal, M. Behari, S.K. Agrawal, The Effect of Step-Synchronized Vibration on Patients With Parkinson’s Disease: Case Studies on Subjects With Freezing of Gait or an Implanted Deep Brain Stimulator, IEEE Trans. Neural Syst. Rehabil. Eng. 21 (2013) 806–811. https://doi.org/10.1109/TNSRE.2013.2250308.

[4] N. Dewan, J.C. MacDermid, Fall Efficacy Scale-International (FES-I), J Physiother. 60 (2014) 60. https://doi.org/10.1016/j.jphys.2013.12.014.

[5] K.O. Berg, S.L. Wood-Dauphinee, J.I. Williams, B. Maki, Measuring balance in the elderly: validation of an instrument, Can J Public Health. 83 Suppl 2 (1992) S7-11.

[6] D. Podsiadlo, S. Richardson, The timed “Up & Go”: a test of basic functional mobility for frail elderly persons, J Am Geriatr Soc. 39 (1991) 142–148. https://doi.org/10.1111/j.1532-5415.1991.tb01616.x.
